# Cyr61/CCN1 targets for chemosensitization in pancreatic cancer

**DOI:** 10.18632/oncotarget.26986

**Published:** 2019-06-04

**Authors:** Snigdha Banerjee, Arnab Ghosh, Daniel D. VonHoff, Sushanta K. Banerjee

**Affiliations:** Cancer Research Unit, VA Medical Center, Kansas City, MO, USA; Department of Pathology and Laboratory Medicine, University of Kansas Medical Center, Kansas City, Kansas, USA

**Keywords:** Cyr61, gemcitabine, pancreatic cancer, chemoresistance

Pancreatic ductal adenocarcinoma (PDAC), commonly acknowledged as pancreatic cancer (PC), is the third leading cause of cancer-related death in the United States, seventh most common cause of cancer-related death globally, and a predicted second-leading cause of cancer-related death by 2030 [1]. Based on the American Cancer Society report (Cancer Facts & Figures, 2019; https://www.cancer.org/content/dam/cancer-org/research/cancer-facts-and-statistics/annual-cancer-facts-and-figures/2019/cancer-facts-and-figures-2019.pdf), more than 56,700 US citizens will be diagnosed with PDAC in 2019. The Surveillance, Epidemiology and End Results (SEER) database estimates an overall five-year survival rate is about 8.2%, which is among the lowest of all solid cancer types. Underlying causes for these depressing results include lack of early detection methods, novel druggable molecules, and limited treatment options [2]. Surgery of course is in option in patients with localized disease. Unfortunately, often the disease comes back after surgery, because, PDAC cells have the propensity to spread to the distant organs in earlier phases of the disease, and these microscopic spreads are non-resectable by surgery.

Cancer immunotherapy is one of the greatest advances in the history of cancer research and treatment [3]. Nevertheless, except for some interesting findings [4–6], immunotherapy in PDAC has not been very useful [7]. Very small percentage of cases where mismatch-repair is presented PD-1 inhibitors can be helpful [8]. Thus, since 1997, gemcitabine (GEM) therapy alone or in various combinations has been one of the standard first-line treatment for patients with unresectable, locally advanced, or metastatic pancreatic cancer, despite having sub-optimal clinical effects with this drug on tumor growth inhibition and the immune system [2, 7, 9]. The sub-optimal effect of GEM is due to weak cellular uptake/activation, poor penetration into the hypo-vascularized and dense tumor stroma (also known as desmoplasia) that all create a barrier for drug delivery [10].

**Figure 1:**
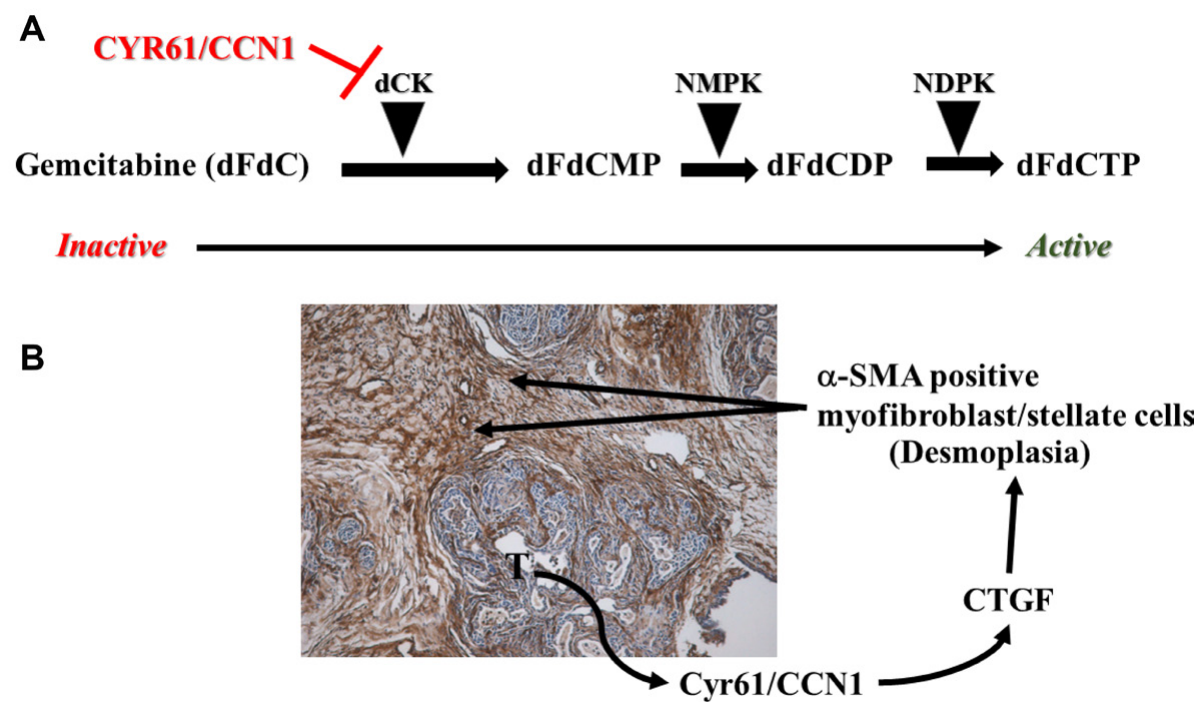
Mechanisms of obstruction of gemcitabine (GEM) delivery in pancreatic cancer. (**A**) Cyr61/CCN1 overexpression results in GEM-inactivation in PDAC cells. Cyr61/CCN1 suppresses dCK expression, which is needed to activate GEM. (**B**) Tumor cell-secreted Cyr61/CCN1 promotes desmoplasia via enhancing CTGF/CCN2 levels in fibroblasts. T, primary tumors; α-SMA, alpha-smooth muscle.

GEM is activated from an inactive pro-drug in cancer cells through a series of phosphorylations by a rate-limiting enzyme deoxycytidine kinase (dCK) and others [11, 12]. PDAC cells can destroy a dCK-pathway and make cancer cells resistant to GEM. Our recent studies found that a matricellular protein CYR61/CCN1, which is overexpressed in PDAC cells and acts as a tumor promoter in PDAC [13], plays a vital role in GEM-resistance via suppressing dCK production in PDAC cells [12] (Figure 1A).

Desmoplasia in PDAC manifest by active myofibroblast/stellate cells and extracellular matrix deposition and a biological barrier to chemotherapy penetration including GEM [14]. Recently, we identified a novel mechanism of regulation of desmoplasia in PDAC. Cyr61/CCN1 is the key player in this novel mechanism. Cyr61/CCN1 promotes and maintains a desmoplastic reaction through activating connective tissue growth factor (CTGF/CCN2)-signaling [12] (Figure 1B). Collectively, these studies suggest that targeting Cyr61/CCN1 in PDAC could be a highly effective in enhancing the sensitivity of GEM.

Given the convincing GEM-resistance-promoting effects of Cyr61/CCN1 as seen in the recent studies [12], there is a reason to be hopeful that multiple mechanisms of GEM-resistance are being disrupted by suppressing the expression of Cyr61/CCN1. Now, we need to find out the molecule that can suppress Cyr61/CCN1 expression in PDAC cells. Furthermore, intense interest is also building around a combination therapy of Cyr61-inhibitor and GEM with immunotherapy. The vital answer to these questions will be forthcoming.
